# The hidden connection between gut microbiota and periprosthetic joint infections: a scoping review

**DOI:** 10.5194/jbji-10-85-2025

**Published:** 2025-03-28

**Authors:** Alessandro Singlitico, Daniele Grassa, Rami Kaplan, Alessandro Smimmo, Giulio Maccauro, Raffaele Vitiello

**Affiliations:** 1 Department of Orthopaedics, Fondazione Policlinico Universitario Agostino Gemelli IRCSS, Università Cattolica del Sacro Cuore, 00168 Rome, Italy; 2 Department of Orthopaedic and Traumatology, Aurelia Hospital Garofalo Healthcare, 00165 Rome, Italy

## Abstract

**Background**: Periprosthetic joint infections (PJIs) pose a significant challenge in orthopedic surgery, and emerging evidence suggests that the gut microbiome may play a crucial role in their development and management. Despite the rarity of these infections, the continuous increase in prosthetic joint arthroplasties has made understanding how to prevent them more pressing. A stronger comprehension of the disruption of the gut microbiome and how this can lead to more of these infections and other pre-surgical risks may be crucial in preventing them. **Objective**: This article aims to provide a stronger understanding of the topic through the analysis of different pieces of already existing literature to help draw new conclusions and raise potential questions that need answering. **Methods**: A comprehensive search strategy without filters was employed, and multiple papers were thoroughly analyzed, understood, and compiled into this paper. **Conclusions**: Despite the limitations of some of the analyzed studies and finite evidence, this paper suggests that there could be a connection between periprosthetic joint infections and a compromised gut microbiome. However, further research is required to draw a definitive conclusion.

## 1 Introduction

Total joint arthroplasty is a safe and effective procedure for improving the quality of life and restoring function in patients affected by end-stage degenerative osteoarthritis (Pulido et al., 2008).

Although periprosthetic joint infection (PJI) is relatively rare, occurring only at a rate of 1 % of all total joint arthroplasties, PJI is one of the most common causes of implant failure and revision surgery (Pulido et al., 2008; Hernandez et al., 2019). Diagnostic criteria for PJI include multiple prerequisites that vary according to the releasing scientific society. The International Consensus Meeting defined major and minor criteria for the diagnosis of PJI, with the major criteria being the following: (1) two identical organisms are found from two separate cultures from the periprosthetic region, (2) the presence of a sinus tract communicating with the joint is determined, and (3) at least three minor criteria from a list are met (includes several tests for elevated leukocyte levels, positive histological analysis of periprosthetic tissue, and elevated inflammation biomarkers) (Parvizi et al., 2013). According to the current literature, there appears to be a propensity for males to develop periprosthetic joint infection after total joint arthroplasty at a higher rate than the female population (De Mauro et al., 2024).

The most common isolated organisms are meticillin-resistant and methicillin-sensitive *Staphylococcus aureus*, along with methicillin-resistant and methicillin-sensitive *Staphylococcus epidermidis* (Toms et al., 2006). The treatment for PJI is challenging, with a success rate ranging from 12 % to 91 % depending on the treatment chosen.

One- or two-stage revision total knee arthroplasty (TKA) is considered the gold standard treatment as it removes the infected, biofilm-contaminated implants and has reported success rates of over 91 % (Rahardja et al., 2023); in debridement, antibiotics, and implant retention (DAIR) the rates of infection control range from 12 % to 80 % (Koyonos et al., 2011).

Moreover, reinfection rates and persistent infection following revision surgery are still as high as 33 %, which also suggests that these patients do not have a protective adaptive immune response (Schwarz et al., 2019).

Traditionally, surgical treatment of PJI has been based on algorithms, where surgical treatment for acute PJI is DAIR, whereas for chronic infection one- or two-stage revision surgery is preferred; recently the system inflammation response index (SIRI) and monocyte-to-lymphocyte ratio (MLR) have been recognized to represent a novel composite index now considered a powerful and reliable indicator of inflammatory conditions (Vitiello et al., 2024).

The total number of joint arthroplasty procedures has risen in the last decade and is expected to grow by 400 % by 2030 (Jenny, 2020). Due to the mortality and morbidity associated with PJI, in addition to the complexity of the treatment, there has been an increased interest in understanding the etiology, pathology, and mechanisms of primary infection. In recent years, there has been a major interest in the role of gut microbiome as a risk factor for PJI. The gut microbiome refers to the microorganisms inhabiting the human gastrointestinal tract (GI tract). The definition of microbiome remains controversial. In fact, microbiome has also been defined more broadly in two ways: as the entire habitat, including the community of bacteria, their genomes, the surrounding metabolites, and so forth, or as the collection of genes and genomes from members of the bacteria (i.e., no viable bacteria) (Schwarz et al., 2024).

Bacteria are by far the most prominent gut-microbiota organisms with five major phyla that account for the majority of bacteria found in the gut: firmicutes, proteobacteria, bacteroides, actinobacteria, and fusobacteria (Guinane et al., 2013). Infections can occur through various mechanisms: direct contact from external contaminants or contiguous spread, hematogenous spread from other body sites, and recurrent infection (Kapadia et al., 2016). Approximately one-third of infections that occur are caused by hematogenous spread from other body sites, which includes any potential gut-microbiota-sourced infection (Rakow et al., 2019). Disruption of a healthy gut microbiome, known as dysbiosis, is associated with an imbalance in the functional interactions of commensal microbes through which either the quantity of beneficial organisms is significantly reduced, the quantity of harmful organisms is significantly increased, or the overall diversity of organisms is reduced (DeGruttola et al., 2016). Dysbiosis may lead to changes in host–microbe interactions, and this is associated with an increased incidence of chronic medical conditions that are also risk factors for PJI, including obesity, diabetes, and inflammatory bowel disease (Al Bander et al., 2020; Knight et al., 2017).

One of the main hypotheses that links PJI and gut-microbiome dysbiosis is the “Trojan horse theory”. It states that organisms from distant sites may be carried from one site to another by leukocyte phagocytosis. In PJI, the ingested microbes might be released into the susceptible periprosthetic area, leading to infection. Other dysbiosis-related factors that may have a link with PJI include increased gut permeability (due to the loss of integrity of the gastrointestinal barrier), inflammatory bowel diseases, and the ingestion of antibiotics (Chisari et al., 2022a).

This scoping review aims to evaluate the connection between the gut microbiome and PJI by analyzing already published articles, identifying gaps, acknowledging what we know, and suggesting potential objectives for future studies.

## 2 Material and methods

In the last few years there has been an increased trend of analyzing the microbiota, in particular gut microbiota and its role in systemic inflammatory response. In fact, within the last 4 years, more than 50 studies were conducted in order to understand the correlation between gut microbiota and its role in PJI. Considering the limited number of studies and the main differences in humans and mice, a scoping review of the literature indexed in the PubMed, MEDLINE, and Cochrane Library databases using search terms “PJI, prosthetic joint infection, microbiome, and microbiota” was conducted. To minimize the number of missed studies, no filters were applied to the search strategy. The bibliography of the selected studies was accurately searched by hand to identify further studies not found during the electronic search by two authors (Daniele Grassa, Raffaele Vitiello). No restrictions were applied concerning the date of publication or the language. The title of the journal, name(s) of the authors, and supporting institutions were not masked at any stage. The searches resulted in 59 articles, and 39 were excluded because they were considered not relevant to the present study. The remaining 20 articles were evaluated in detail to verify their congruence with the inclusion criteria. Inclusion criteria were studies describing the animal and murine in English. References of relevant papers were then analyzed to find additional works pertinent to the topic. Abstracts and full texts were independently screened by four authors (Alessandro Singlitico, Daniele Grassa, Rami Kaplan), and any discordance was resolved by consensus with a fifth author (Raffaele Vitiello). Each article was evaluated by three independent investigators (Alessandro Singlitico, Daniele Grassa, Rami Kaplan). After full text screening, a total of four studies were selected meeting the inclusion criteria and a scoping review was conducted.

## 3 Discussion

Many studies have been conducted in order to research the role of gut microbiota in PJIs.

In 2019, Christopher J. Hernandez analyzed a mouse model to observe whether or not the state of the gut microbiota before surgery influences the local and systemic responses against infection (Hernandez et al., 2019). In this experiment, mice were divided into two different groups: those with unmodified gut flora and those with modified gut flora using antibiotics (Δmicrobiome group) (Hernandez et al., 2019). Additionally, they received a titanium tibial component to mimic a joint implant and a local synovial inoculation of *S. aureus* was conducted to establish infection. A total of 5 d later noninvasive signs of infection were observed (radiographs, gait score, body weight, and fecal pellet) (Flannagan et al., 2016) as well as systemic inflammatory response aspects (serum amyloid A and T-cell population in the lymph nodes and spleen) (Hernandez et al., 2019). The study showed that the number of subjects in the Δmicrobiome group which developed PJI was greater, had a poorer gait score, a greater reduction in body weight after surgery, and higher levels of serum amyloid A, which is associated with a greater bacterial load in surrounding joint tissues, demonstrating that the composition of the microbial community of Δmicrobiome is different.

In 2020, Zhu et al. (2020) conducted an animal study on rats to test the Trojan horse theory (Flannagan et al., 2016). A total of 40 adult rats were colonized intestinally with a green fluorescent protein (GFP)-tagged methicillin-resistant *Staphylococcus aureus* (MRSA), and to simulate a prosthetic joint implant, a Kirschner wire (K-wire) was used and inserted in the knee joint. The K-wire was inserted in their knee joints either at 8 or 72 h after MRSA colonization (n=20 each). Mice were euthanized at 10 d, and tissues such as the capsule, the femur, and the implanted K-wire were harvested and collected for analysis of PJI. The intraoperative cultures from all 40 rats were negative, indicating the absence of intraoperative contamination. In the 8 h group, 25 % of rats developed PJI, while in the 72 h group, 10 % of rats did. The experiment was repeated with MRSA colonization using isolates taken from PJI patients, where 30 % of rats in the isolate 8 h group developed PJI and 35 % in the isolate 72 h group did. As a control for the above, a group of mice had the K-wire inserted without colonization. None of the mice in this control group developed PJI. These results suggest that colonized MRSA originating from the gut can cause infections at distal surgical sites in the operated knee joint with an intra-articular implant (Zhu et al., 2020).

A similar study conducted by Chisari et al. (2022b) investigated whether or not many PJIs can emerge from endogenous sources of microorganisms, especially the gut microbiome. The study focused on the interaction between gut dysbiosis and gut permeability using Zonulin as a marker of intestinal permeability, as well as soluble CD14 (sCD14) and lipopolysaccharides (LPS) as indicators of inflammation and bacterial translocations (Fasano et al., 2000; Wang et al., 2000; Fasano, 2020). The study hypothesized that the levels of Zonulin, LPS, and sCD14, known indicators of gut permeability, were associated with PJI, indicating that some degree of gut dysbiosis may be at play in patients who develop PJI. The study illustrated that the levels of Zonulin and sCD14 were found to be elevated in PJI, with Zonulin levels higher in acute PJI compared to chronic PJI (Chisari et al., 2022b), strengthening the hypothesis that the gut microbiome and permeability have a role in shaping immune response.

Gut permeability alteration was the main focus of another study conducted by Chisari et al. (2022c) in the same year to examine how bacteria from the intestinal site can translocate due to a higher permeability as seen in patients with inflammatory bowel disease (IBD) (Aleksandrova et al., 2017; Halfvarson et al., 2017; Li et al., 2017). A total of 152 consecutive primary arthroplasties (130 total hip arthroplasties and 22 total knee arthroplasties) performed in patients with a diagnosis of IBD were identified. Patients with IBD had a higher rate of PJI at 2 years postoperatively, as well as a higher rate of complications and aseptic revision. It was hypothesized that patients with a disrupted gut membrane, such as those with IBD, may experience dysbiosis that can translate to increased infectious complications (Chisari et al., 2022c).

## 4 Limitations of the study

This scoping review has some limitations due to the quality of the studies included. The main limitation of this review is the fact that only two animal studies and two human studies were available. The only two human studies were performed in the same institute by the same authors in the same year and with the same cohort. In addition, both studies in murines do not include sufficient randomization and blinding, as both the researchers and the assessors of the results were not prevented from knowing into which group the murines in Table 1 were placed. Furthermore, the studies had different protocols for moderating the risk of bias. In fact, the animal studies scored rather low on the SYRCLE test, having an increased risk of bias. Moreover, even with high-scoring studies, the risk of omitted/missing data has to be considered, and this can be more relevant in narrative reviews where a limited number of studies are selected. To conclude, all of the studies were written on different types of dysbiosis, so it is difficult to individualize the connection between the gut microbiome and the risk of PJI.

**Table 1:** Characteristics and findings of included studies.

Authors	Date	Type of	Dysbiosis	No. of	Length	Analyzed	Results
	published	study	type	patients/	of	variables	
				subjects	follow-		
					up		
Hernandez	Jul 2019	Mouse study	Altered gut	82	5 d	Bacterial load	Higher PJI occurrence in
et al. (2019)			microbiota			(CFU) on	altered gut microbiota mice
			due to			implant	vs. control (72.5 % vs. 50 %,
			antibiotics			surface;	p=0.03) and lower
						Shannon	microbiome diversity
						diversity	(2.36±0.57 vs. 3.87±0.28,
						index for	p<0.001)
						microbiome	
						diversity	
Zhu et al.	Apr 2020	Preclinical –	Intestinal	100	10 d	Stool analysis	MRSA strain: 25 % of rats in
(2020)		rat study	MRSA			on days 1–10;	the 8 h group and 10 % of rats
						after sacrifice,	in the 72 h group developed
						specimens from	PJI; MRSA isolate: 30 % of
						knee joint,	rats in isolate 1 and 35 % in
						femur, and	isolate 2 developed PJI;
						implanted K-wire	control: 0 rats developed PJI
						harvested and	
						cultured for	
						bacterial	
						identification;	
						16S ribosomal	
						DNA, GFP gene	
						expression,	
						and PCR used	
						for analysis	
Chisari et	Sep 2022	Prospective	IBD—gut	134	n/a	Zonulin, LPS,	CD14 and Zonulin higher in
al. (2022b)		cohort study	permeability			sCD14	PJI vs. non-PJI patients
						(markers of	(555±216 ng mL^−1^ with p<0.05
						gut	and 7.642±6.077 ng mL^−1^ with
						permeability)	p<0.001, respectively); Zonulin
							higher in acute PJI group vs.
							chronic (10.7±6.2 ng mL^−1^ vs.
							
							5.8±4.8 ng mL,
							p=0.005)
Chisari et	Jan 2022	Retrospective	IBD	608	2 years	PJI cases in	IBD group more likely to
al. (2022c)		cohort study				IBD patient	develop PJI vs. non-IBD
						group and	control (4.61 % vs. 0.88 %,
						non-IBD group;	p=0.0024);
						analyzed using	IBD associated with
						univariable	5.4-fold higher incidence of
						regression	PJI (p=0.007)
						with a Cox	
						regression	
						model	

n/a means not available.

Hernandez et al. (2019) focused the study on discovering a link between reducing gut diversity and an increased risk of PJI. Firstly, the gut microbiota was modified in growing mice, so the possibility that the findings of the study were influenced by early changes in the development of the host's immune system cannot be ignored. Alterations in the gut microbiome at later ages may have different effects on the response to bacterial challenge. Secondly, there are limitations in extrapolating findings to humans, including differences between the two species regarding the constituents of their microbiomes (Jandhyala et al., 2015) and immune systems (Chisari et al., 2022c). Thirdly, the current study only examined male mice, and the response may differ in females. Lastly, the constituents of the microbiome were modified using chronic oral antibiotics such as neomycin and ampicillin, which respectively have zero or negligible bioavailability in rodents (Manzanares et al., 2016), suggesting that trace amounts may have been distributed in the animals.

Looking at the murine studies, Zhu et al. (2020) concluded that intestinal MRSA could cause PJI (Zhu et al., 2020). The main drawback of this study is that it was an observational cohort study, whereas the human studies were structured with a control group that included healthy and infected populations. The results suggested that the Trojan horse theory could be linked to gut infections, and this is important as MRSA-caused PJI tends to be more severe than PJIs caused by other microorganisms (Cunningham et al., 2017).

First, rats have a different immune response than humans, so the applicability of our results to humans is limited, and even though the Trojan horse mechanism has been well described in human cells (Flannagan et al., 2016), more clinical evidence to support the actuality of this mechanism, particularly evidence related to PJI, must be evaluated. Secondly, the study focused on neutrophils as being the Trojan horse not considering that monocytes, considering they are less than 5 % of white blood cells in rats, or other non-phagocytic immune cell types could also be involved in this Trojan horse mechanism. Finally, the bacterial burden of MRSA in the gut was too high and clinically the virulence of intestinal MRSA is expected to be significantly lower.

The study by Chisari et al. (2022b) examined gut permeability, which can may play a role in the risk of developing a PJI (Chisari et al., 2022b). The limitation of this study is the fact that it is based on a single-center patient population, and its generalizability may be limited. Additionally, all the markers examined are proteins that behave as acute phase reactants, and their levels have been shown to correlate with other biomarkers of inflammation (Fasano et al., 2000; Sánchez-Alcoholado et al., 2020). Finally, the biomarkers examined are indirect measures of gut epithelial barrier integrity, and direct measurement of gut dysbiosis and inflammation would require invasive testing, which is not performed. However, this approach seems scientifically reasonable as many previous studies have relied on indirect measures of gut dysbiosis to implicate its relationship with various diseases (Fasano et al., 2000; Fasano, 2020; Arrieta et al., 2006; Turner, 2009; Ciccia et al., 2017; Li et al., 2016).

Finally, the second study of Chisari et al. (2022c) analyzed the human studies regarding the risk of developing PJI in IBD (Chisari et al., 2022c) and control patients. Patients included in this study were identified using the International Classification of Diseases (ICD) codes, with high risk of some patients with IBD being unnoticed. Second, the sample size was relatively small, which put the study at risk for a type II error. Another limitation of the study was that it was not able to shed light on the exact cause of the higher observed PJI and aseptic failure rates in patients with IBD. Although the increase in treatment failure seen in the patients with IBD may stem from their underlying autoimmune status and the inflammatory process in the gastrointestinal tract, malnutrition may have also played a role and a recent meta-analysis showed a smaller effect of malnutrition on PJI (Tsantes et al., 2019); therefore, IBD alone may be the main culprit here. Finally, detailed data regarding the duration or the severity of IBD in this cohort were unavailable.

In non-IBD patients, dysbiosis could be better managed, and patients may benefit from gut permeability and inflammation marker analysis pre-operatively. Research should also target the complete characterization and treatment of the “leaky gut”, its potential reversal due to prebiotics and probiotics, and how the incidence rate of developing PJI can be modified. The leaky gut is not fully understood and seems to occur due to increased intestinal permeability caused by various factors including endurance exercise, liver diseases, esophagitis, neurological diseases, psychiatric diseases, food allergies, altered gut microbiota (as seen in this review), altered metabolism, aging, and pharmaceutical intervention aimed at the gut. Its current methods of measurement include fractional urinary excretion of orally administered probe molecules (in vivo and in vitro using a mucosal biopsy) and endoscopic measurements of the intestinal barrier, but none of the current methods are universally accepted (Iannotti et al., 2020). Thus, future research on the measurement protocols can be helpful in both understanding the role of the gut bacteriome in PJI and potentially finding a commonly accepted measure of a leaky gut. The usage of prebiotics to treat IBD showed limited efficacy, and their usage in non-IBD patients has no contraindications (Sartor and Wu, 2017). Finally, it is important to consider the role of other commensal gut microbiota such as fungi (Jovic et al., 2022). Fungal infections of PJI, although rare (accounting for <2 % of PJI cases), represent a medical challenge as they currently have a poor prognosis and many treatment-resistant strains since they occur in cases of immunosuppression (Pérez, 2021). Unlike fungi and bacteria, PJI has not been found to be caused by other microorganisms such as archaea, protists, and algae (Wu and Schmitz, 2023); however, these other microorganisms could still influence the risk of PJI when there is a state of dysbiosis in the gut since Hernandez et al. (2019) described the fact that the reduction of the overall diversity of microbiota was associated with an increased risk of PJI.

## 5 Considerations and future investigations

These findings are clinically relevant, calling our attention to the role that gut modulators such as probiotics and prebiotics may play in the management of patients with PJI who are routinely subjected to prolonged courses of antimicrobials. Future studies exploring the “gut-immune-joint axis” and the role that modulators of gastrointestinal may play are needed. Currently, the most important consideration in the medical field is primary prevention in order to reduce infection risk during joint replacement. Natural barriers, such as skin and nasal mucosae, can act as barriers to infection, and their decolonization before surgery from known pathogens may reduce potential sources of infection (Benito et al., 2019). The GI tract represents another crucial barrier and when altered by diseases such as HIV, celiac disease, and irritable bowel syndrome may increase the risk of PJI (Sartor and Wu, 2017). In addition to the prevention of gut dysbiosis, it is necessary to treat and counteract chronic diseases that may alter GI permeability; to mitigate the impact of gut dysbiosis on PJI, probiotics and prebiotics can be considered to reduce the risk of complications (Manzanares et al., 2016). Another point that has to be investigated is whether antibiotic therapy affects host immunity to musculoskeletal infection (MSKI). It must also be considered that every patient may have a different microbiome milieu, and the effects of the antibiotic therapy can vary between individuals, as can its influences on the immune system. In fact, antibiotic therapy studies in germ-free mice to control the effect of the gut microbiome or “omics” studies such as mass spectroscopy or RNA sequencing to understand the proteomic and transcriptional landscape altered by antibiotic therapies have to be conducted. Moreover, comparisons between different antibiotic types to determine whether there is specificity in an altered host immunity depending on the type of antibiotic used and how these affect patients with different microbiome milieus according to life style, diet, health status, and gender have to be made (Schwarz et al., 2024). The field has potential for a scientific breakthrough in orthopedic research, given the positive results displayed in the data. Firstly, more research should be performed in mice and rat models to fully understand if there is a connection between PJI risk and the main types of dysbiosis, including Crohn's disease and ulcerative colitis. The characterization of the types of leukocytes that are most likely to act as “Trojan horses” should be investigated so that future research can be focused on prevention and treatment. Given that IBD patients show an increased risk of PJI, research in humans should target how to reduce the risk of infection in these populations, analyzing the risk of developing both acute (≤90 d) and chronic PJI (>90 d) and considering whether there is a difference in those two scenarios. If this should be demonstrated, patients may benefit from surgeries scheduled when IBD is mitigated in order to reduce the chances of leukocytes acting as Trojan horses. To better investigate the Trojan horse theory, it will be interesting to determine the best methods for classifying and detecting leukocytes with techniques such as single-cell sequencing. In single-cell sequencing, the samples are analyzed for their RNA content and converted to cDNA, which can then be amplified to find traces of existing or broken-down leukocytes (Jovic et al., 2022), indicating the type that was present – the infection vector could be a single leukocyte after all. Furthermore, the analysis of the role of inflammatory blood cells associated with inflammatory blood markers such as SIRI and MLR could be helpful in predicting a favorable outcome in the case that a prosthetic explant for PJI could be predictive of a favorable outcome. The evaluation of these laboratory indices, especially their determination at 4 weeks after removal, could therefore help to determine which patients could be successfully replanted and to identify the best time to replant. More studies analyzing a wider cohort of patients with chronic PJI are needed to validate the promising results of this study (Vitiello et al., 2024). To conclude, although there have been several studies demonstrating the potential of anti-biofilm monoclonal antibodies (mAb), experimental evidence proving that mAb can eradicate biofilms in animal models remains to be formally demonstrated. This is partly due to the limited resolution of in vivo outcome measures to make such a bold and definitive conclusion. Thus, research funding is needed to enhance both the sensitivity and specificity of biofilm eradication measurements and the evaluation of promising mAb in validated animal models of MSKI with quantitative outcome measures of their biofilm infection (Schwarz et al., 2024).

## 6 Conclusions

To conclude, the results of the above studies describe the fact that gut dysbiosis does increase the risk of PJI in both murines and humans, even if there is limited evidence. PJI patients were associated with altered gut permeability and an elevation in inflammation markers, suggesting an underlying dysbiosis. This is associated with an increased risk of PJI caused by altered gut biodiversity, the presence of intestinal inflammatory bowel diseases, increased gut permeability, or an alteration in the healthy gut microbiota ratio. Future research should focus on preventing dysbiosis and understanding when this should be treated.

## Data Availability

Data were manually extracted from published data in studies referenced in this review. A further request to access data can be made by sending an email to the corresponding author.

## Code availability

The code used for data analysis is not publicly available but can be provided upon reasonable request to the corresponding author.
